# Mechanical Properties of Fiber-Reinforced Permeable Geopolymer Concrete

**DOI:** 10.3390/ma16176030

**Published:** 2023-09-01

**Authors:** Lina Xu, Qilong Liu, Xu Ding, Shuang Sun, Zhanfang Huang

**Affiliations:** 1 School of Transportation Science and Engineering, Jilin Jianzhu University, Changchun 130118, China; xulina@jlju.edu.cn; 2 School of Civil Engineering, Jilin Jianzhu University, Changchun 130118, China; liuqilong1997@outlook.com (Q.L.); dingxu0902@outlook.com (X.D.); 3 School of Architectural Engineering and Space Information, Shandong University of Technology, Zibo 255049, China; huangzhanfang@163.com

**Keywords:** geopolymer, permeable concrete, steel fiber, permeability, unconfined compressive strength

## Abstract

In this paper, permeable geopolymer concrete with high compressive strength and permeability is prepared using alkali-activated metakaolin as a slurry, and its mechanical properties are reinforced by adding steel fibers. The influencing factors of the strength, porosity and permeability coefficient of the fiber-reinforced permeable geopolymer concrete, as well as its microstructure and curing mechanism, are determined by conducting an unconfined compressive strength test, scanning electron microscopy, energy-dispersive spectroscopy and X-ray diffraction. The test results show that, under the water permeability required to meet the specification conditions, when the alkali activator modulus is 1.4 and the activation-to-solid ratio is 0.9, the effect of metakaolin activation is the most obvious, and the unconfined compressive strength of the permeable geopolymer concrete is the highest. Moreover, the paste formed via the alkali activation of metakaolin contains a large number of silica–oxygen and aluminum–oxygen bonds with a dense and crack-free structure, which enables the paste to tightly combine with the aggregates; the strength of the permeable geopolymer concrete is early strength, and its strength at a curing age of 3 days is the highest. The strength at a curing age of 3 days can reach 43.62% of the 28-day strength; the admixture of steel fiber can effectively improve the strength of the permeable concrete, and with an increase in the amount of admixture, the strength of the fiber shows a trend of increasing, and then decreasing. Under the conditions of this test, a volume of steel fiber of 0.3% enables the optimum unconfined compressive strength to be reached.

## 1. Introduction

Cement is one of the most widely used construction materials, but its production process generates a large amount of carbon dioxide [1]; therefore, there is an urgent need to find suitable substitutes to help the construction industry realize the goal of “double carbon”. Geopolymers have been developed in recent years as a new type of cementitious material with excellent performance [2]. Traditional permeable concrete mainly consists of silicate cement, coarse aggregate, admixture and water, and according to its performance design, a small amount of fine aggregate and mineral admixture can be added; however, there are problems with this type of concrete, such as low strength and poor durability after hardening [3]. Alkali-activated geopolymer cementitious materials are not only environmentally friendly but also have excellent engineering performance; therefore, they are regarded as advanced green cementitious materials with excellent performance that can replace the cement matrix and have great development potential [4]. Compared with the cement production process, the geopolymer production process does not require high-temperature calcination and involves no decomposition of carbonate minerals; moreover, its CO_2_ emissions are only 1/10 to 1/5 of that of cement production [5], and its energy consumption is only 1/6 to 1/4 of that of cement production [6].

To date, domestic and foreign scholars have carried out research work on geopolymer gelling materials. Wang Aiguo et al. [7] obtained geopolymer cementitious materials with a 3-day strength of 115 MPa after activation of the product of the high-temperature calcination of coal metakaolin by an alkali activator. Siddique and Kaur [8] tested concrete made by replacing the cement with 0 wt.%, 5 wt.%, 10 wt.% and 15 wt.% metakaolin, and found that the compressive strength of the concrete reached its maximum following replacement with 10 wt.%. Duan et al. [9] tested concrete made of metakaolin, silica fume and blast furnace slag and found that the mechanical properties of concrete made with metakaolin were better. Bernal et al. [10] found that alkali-activated slag exhibits a rapidly hardening phenomenon, which can be inconvenient in actual field construction, but the addition of metakaolin to an alkali-activated slag system can efficiently improve this phenomenon. Fu et al. [11] conducted tests on cement, cement + silica fume, alkali-activated slag and three kinds of permeable slurry concrete, and found that slag activated by an alkali activator can improve the unconfined compressive strength of permeable concrete and resistance to sulfate erosion. Jo et al. [12] studied fly ash and nano-SiO_2_ following the alkali activator activation of permeable geopolymer concrete, and determined their mechanical properties with the activation-to-solid ratio, gelling material, sodium hydroxide concentration, etc. Chang et al. [13] combined slag with porous coarse aggregate after activation by an alkali activator to make permeable concrete with a 28-day strength of more than 35 MPa and a permeability coefficient higher than 4.9 mm/s. Sun et al. [14] showed that permeable geopolymer concrete prepared using slag, metakaolin and an alkali activator resulted in better mechanical and permeability properties. The compressive strength of permeable geopolymer concrete obtained using alkali-stimulated high-calcium fly ash via THO [15] can reach more than 10 MPa with a void ratio of 30%. Za [16] used bottom ash to make a coarse aggregate of permeable concrete with alkali-activated fly ash as the cementitious material and found that the higher the concentration of sodium hydroxide, the higher the concentration of silica–aluminum ions dissolved, and the higher the degree of the reaction of polymerization.

After hardening, traditional permeable concrete has low strength, poor durability and other problems [3], and compared to ordinary silicate concrete, after hardening, geopolymer concrete has high strength and good resistance to acid, alkali and salt corrosion, but its brittleness is more pronounced. A study showed that the addition of steel fibers can improve the mechanical properties of concrete. Fang et al. [17] selected 6 mm- and 12 mm-long basalt fibers for testing, and found that compared to a vegetative permeable concrete fiber length of 12 mm, with a volume dosage of 0.1 wt.%, when the compressive strength increased by 15.48%, the flexural strength increased by 110%. Tao et al. [18] pointed out that an increase in steel fiber content will enhance the mechanical properties of geopolymer concrete, such as its strength, modulus of elasticity and ductility. Kumar et al. [19] found that steel fibers can effectively delay the expansion of cracks and improve the fracture energy and ductility of the material. Farhan et al. [20,21] investigated the mechanical properties of geopolymer concrete reinforced with steel fibers and carried out a uniaxial compressive stress–strain full-curve test, and found that the compressive, tensile and flexural strengths, as well as the post-peak ductility, of the full curve of steel fiber concrete increased with an increase in steel fiber content. Wang et al. [22] found that the optimum unconfined compressive strength of permeable concrete was achieved by admixing 90–120 g of steel fibers in 100 mm × 100 mm × 100 mm specimen blocks. Fiber content also affects water permeability. Jiang Kuan et al. [23] mixed steel imitation fibers into permeable concrete and found that the fibers reduced the water permeability, but with an increase in fiber content, the water permeability instead increased. Gabriel et al. [24,25] incorporated wood fibers into fly-ash-based geopolymer composites and found that they reduced the density of the composites while elevating the conformal material loads.

The current research mainly focuses on the mechanical properties of geopolymer concrete and traditional permeable cement concrete. There has been little research on the preparation of permeable geopolymer concrete using geopolymers instead of cement as the cementitious material; thus, the present study determines the strength and water permeability properties of permeable geopolymer concrete through an unconfined compressive strength test, a water permeability test, energy-dispersive spectroscopy and X-ray diffraction, based on which we can improve the mechanical properties of permeable geopolymer concrete through the addition of steel fibers to determine the optimal amount of steel fibers. In this paper, alkali-activated metakaolin is used as a cementitious material instead of cement to prepare permeable geopolymer concrete. While meeting the requirement of water permeability, steel fibers are added to increase the strength of the permeable geopolymer concrete. In addition, the use of metakaolin, a form of industrial waste, as a substitute for cement illustrates the feasibility of the comprehensive utilization of industrial waste and reduces carbon emission, making it environmentally friendly.

## 2. Test Materials and Methods

### 2.1. Test Materials

**Figure 1:**
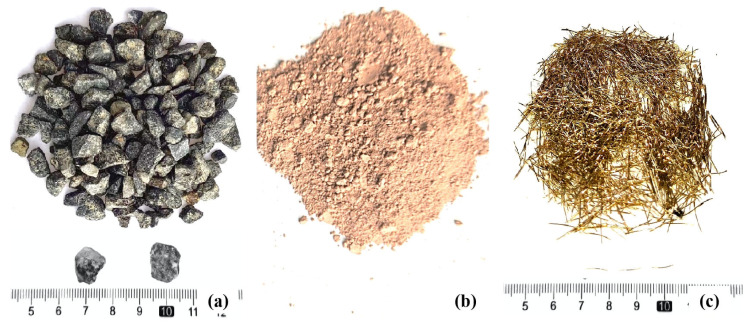
Raw test materials. (**a**) Coarse aggregate (**b**) Metakaolin (**c**) Steel fiber.

Coarse aggregate with a particle size of 4.75~9.5 mm was purchased from Zhejiang Borui Co., Ltd., Wenzhou, China, with a compact packing density of 1530 kg/m^3^, as shown in Figure 1a. Metakaolin (Figure 1b) with a fineness of 1250 mesh and an activity index of no less than 110 was purchased from Wanying Environmental Protection Materials Co. Ltd., Gongyi City, Henan Province, China, and its basic properties are shown in Table 1. Sodium hydroxide with an analytical purity of 96 wt.% or more was purchased from Jilin Hengji Co., Ltd., Changchun, China. The modulus of soluble glass is 3.3, purchased from Yurei Co., Ltd., Shanghai, China, and the basic properties are shown in Table 2. Steel fiber (Figure 1c) with a length of 8~10 mm and a diameter of 0.18~0.23 mm was purchased from Hebei Baihang Engineering Co. (Handan, China). In the test, considering the adaptability of water-reducing agents and alkali-excited materials, we chose a naphthalene water-reducing agent, purchased from Henan Hengtai Environmental Protection Company, Zhenzhou, China, with a dosage of 1 wt.%; its performance index is shown in Table 3.

This test specimen was made using a volumetric method; the porosity was determined first, and then the specimen material mix ratio was calculated according to Equation (1):

(1) $$\frac{m_{c}}{\rho_{c}}+\frac{m_{f}}{\rho_{f}}+\frac{m_{g}}{\rho_{g}}+\frac{m_{w}}{\rho_{w}}+\frac{m_{a}}{\rho_{a}}+P=1$$

where *m* is the mass of each material, *p* is the apparent density of the material, *P* is the design porosity, *c* is the fiber, *f* is the metakaolin, *g* is the coarse aggregate, *w* is the alkali activator solubilization and *a* is the water reducer.

### 2.2. Sample Preparation

**Figure 2:**
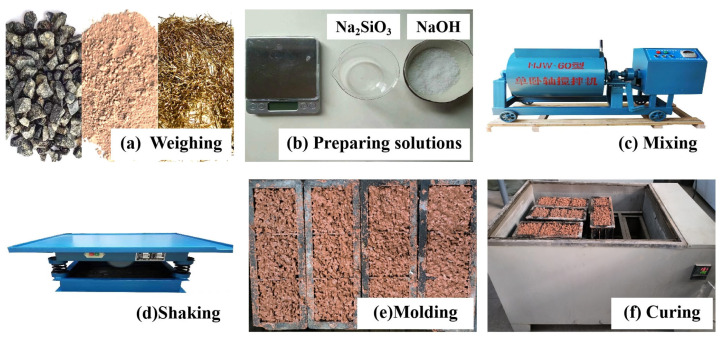
Specimen preparation and curing process.

The specimen preparation process included the weighing of raw materials, solution preparation, mixing, molding, vibration, standard maintenance and other steps. The process is shown in Figure 2, and the specific steps are as follows.

(1) Preparation stage: The aggregate is passed through a sieve with a pore size of 5 mm to remove extra sediment and impurities for spare parts. The alkali activator solution is configured 24 h in advance to ensure that the sodium hydroxide particles are fully dissolved in the sodium silicate solution, and then placed into a thermostat box for preservation after cooling to room temperature.
(2) Production stage: First, a single horizontal-axis mixer is used to mix the aggregate and metakaolin for 30 s, so that the metakaolin powder evenly wraps around the aggregate; then, it is slowly poured into the alkali stimulant solution and mixed until no dry powdery metakaolin remains; and finally, the solution is poured into 100 mm × 100 mm × 100 mm molds, and the molds are placed on a shaking table where they undergo vibration for 9 s, and the surface of the test block is finally smoothed.
(3) Maintenance stage: The test block is placed under outdoor maintenance for 24 h, and then placed into the maintenance box (temperature: 20 ± 2 °C/humidity: 98%) for steam maintenance 3 days after demolding, where it remains until the specified age.

### 2.3. Test Methods

(1) Unconfined compressive strength test

The unconfined compressive strength test was performed using the microcomputer servo universal testing machine produced by Jilin Guanteng Automation Technology Co., Ltd., Changchun, China, which adopted uniform displacement speed control and a loading speed of 0.1 mm/s, and the load and deformation were automatically collected by the system. Three test blocks per batch were tested according to the standard and the results were averaged. According to the standard deviation, if there is a large dispersion of data, we should discard the data and re-select the test block for the test.

(2) Determination of permeability coefficient

**Figure 3:**
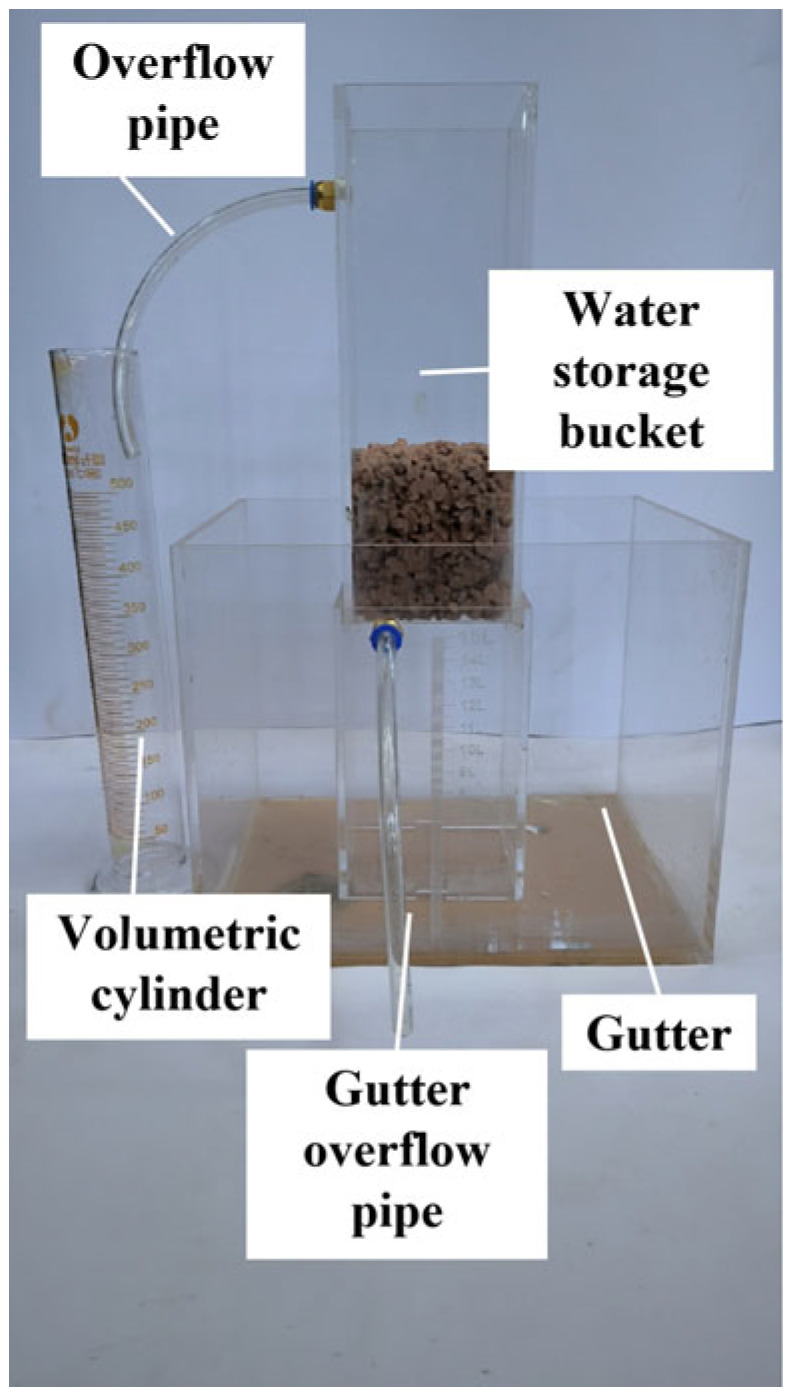
Permeability meter.

We placed the specimen into a water permeability meter, as shown in Figure 3, sealed the outside evenly using rubber cement, and then carried out a sealing check. After the sealing check was conducted, the water permeability measurement began. Three test blocks per batch were tested according to the standard and the results were averaged. According to the standard deviation, if there is a large dispersion of data, we should discard the data and re-select the test block for the test. The water permeability coefficient *K* can be calculated using Equation (2).

(2) $$K=\frac{QL}{AHt}$$

where *K* is the permeability coefficient (cm/s); *L* is the thickness of the specimen (cm); *H* is the difference in water level, i.e., the height difference between the mouth of the overflow pipe and the mouth of the outlet pipe (cm); *Q* is the amount of exudate water in *t* seconds (mL); and *A* is the cross-sectional area of the specimen (cm^2^).

(3) Porosity Determination

The porosity determination of the permeable concrete was carried out using the mass method. We soaked the specimen in water for 24 h to saturate it with the mass of the specimen in water *W*_1_, removed the specimen, placed it in the air to dry, and when the mass was constant (after about 24 h, depending on the indoor and outdoor temperatures), weighed the mass of the specimen in the air *W*_2_ and the volume of the specimen *V*. Three test blocks per batch were tested according to the standard and the results were averaged. According to the standard deviation, if there is a large dispersion of data, we should discard the data and re-select the test block for the test. The total porosity of permeable concrete *n_e_* can be determined according to Equation (3):

(3) $$n_{e}\;=\;1-\frac{W_{2}-W_{1}}{V}*100\%$$

(4) Scanning electron microscope and energy-dispersive spectroscopy analysis

Scanning electron microscopy (SEM) and energy-dispersive spectroscopy (EDS) were performed using a scanning electron microscope manufactured by Carl Zeiss Ltd., Shanghai, China.

(5) X-ray diffraction analysis

X-ray diffraction analysis using an X-ray diffractometer from Rigaku, Tokyo, Japan, only extracted, from the permeable geopolymer concrete slurry part of the ground powder placed in the X-ray diffractometer, the alkali activation of metakaolin products for the determination of the chemical composition.

## 3. Test Results and Analysis

### 3.1. Effect of Alkali Activator Modulus on Permeable Geopolymer Concrete

**Figure 4:**
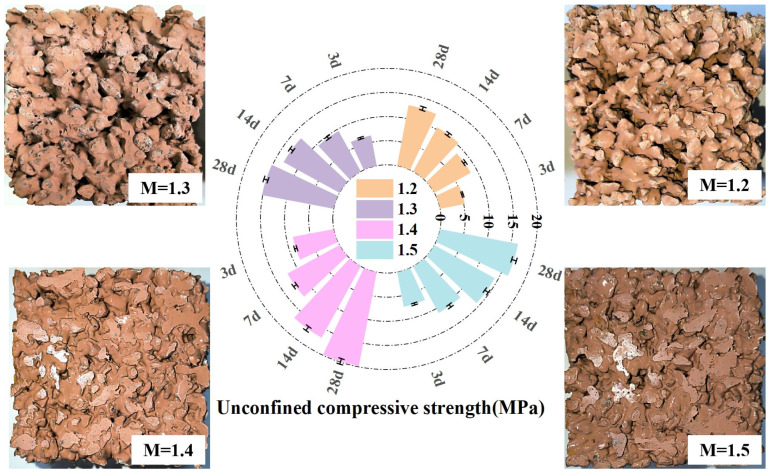
Relationship between solution modulus and unconfined compressive strength.

Figure 4 shows the relationship between the solution modulus and the unconfined compressive strength. As can be seen from Figure 4, the unconfined compressive strength of the permeable geopolymer concrete increases with an increase in the age of curing; however, with an increase in the modulus of the alkali activator solution, the strength of the permeable geopolymer concrete undergoes a process of increasing, and then decreasing, when the modulus of M = 1.4, the point at which the activation of metakaolin has the greatest effect. When the alkali activator modulus is low, the solution is easily solidified, and when the mix is too dry and hard after mixing, the specimen is not easily formed. When the alkali activator modulus is high, the solution has fewer oligosiloxane tetrahedral groups, which is not conducive to the reaction, and when the mix is too dilute, the specimen strength after molding is low. When the modulus M = 1.4, the slurry can be more uniformly adhered to the aggregates.

### 3.2. Effect of Activation-to-Solid Ratio on the Strength of Geopolymerized Permeable Concrete

**Figure 5:**
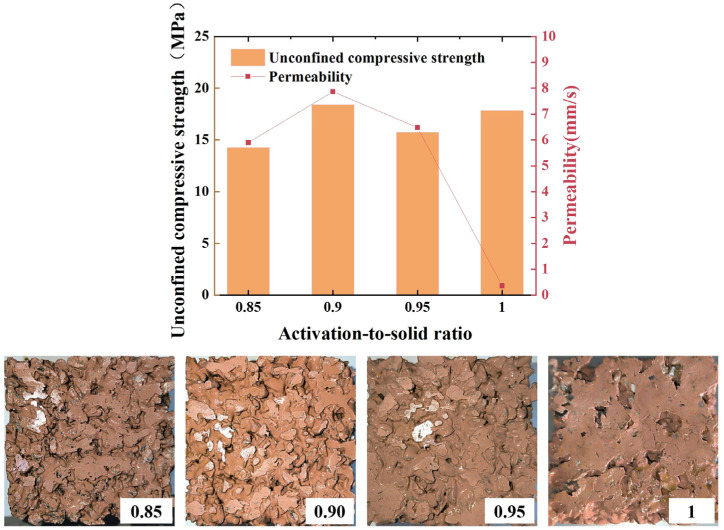
Plot of activation-to-solid ratio versus unconfined compressive strength and permeability coefficient.

Figure 5 shows the relationship between the activation-to-solid ratio and the unconfined compressive strength and water permeability coefficient. As can be seen from Figure 5, the strength curve of permeable geopolymer concrete shows a trend of first increasing, and then decreasing, and finally, increasing; this is because when the excitation-to-solid ratio is lower, there is a large gap between the quality of the alkali excitant and the quality of metakaolin. Metakaolin cannot be completely mixed with the alkali activator solution, and therefore, it cannot be completely activated to give full play to its due activity, so its strength is low. When the activation-to-solid ratio is 0.9, the metakaolin is completely activated, the specimen has the maximum unconfined compressive strength, and its permeability coefficient is the largest. As the excitation-to-solid ratio continues to increase, the permeable geopolymer concrete strength decreases, because when the excitation-to-solid ratio is too high, the quality of the alkali activator is close to the quality of metakaolin, although the metakaolin is fully activated; however, at this point, the formation of the cementing slurry is too dilute to enable good mobility, meaning the slurry cannot be uniformly wrapped around the aggregate, resulting in a reduction in strength. The intensity of the excitation-to-solid ratio increases to 1 instead of decreasing, which is a result of the decrease in strength. Because the dilute slurry after vibration can easily form a sealing slurry at the bottom, the previous pore structure of the permeable geopolymer concrete changes into a dense structure so that the strength becomes high, but at this point, the test block almost loses its permeability. When the excitation-to-solid ratio is 0.9, the aggregate is uniformly wrapped by the slurry, there is no water seepage and the permeability coefficient meets the specification requirements.

### 3.3. Compressive Strength Prediction Model Based on Griffith Fracture Theory

At present, the compressive strength prediction models based on pore structure characteristics are mostly expressions of the relationship between porosity and compressive strength, and demonstrate the effect of the percentage of pore space on the compressive strength of permeable concrete; these prediction models mostly involve simple regression fitting, and lack theoretical support. The Griffith fracture theory is often used as a classical theory to describe the relationship between the strength and porosity of solid brittle materials [26], and its expression is as follows:

(4) $$\delta_{max}=\sqrt{\frac{2E\gamma }{\pi \alpha }}$$

where $\delta_{max}$ is the ultimate stress at damage (N/m^2^); *E* is the modulus of elasticity (N/m^2^); *γ* is the material surface fracture energy (J); and *α* is half of the initial crack length (m).

Permeable concrete’s pores do not provide an elastic modulus, but there is a certain functional relationship with the elastic modulus. Studies have been conducted to establish the effective elastic modulus of porous materials as a function of porosity, and it has been found that the elastic modulus decreases as porosity increases. In this paper, the relationship between the two is expressed by the following equation:

(5) $$E=E_{0}e^{-m_{0}P_{T}}$$

where *E*_0_ is the elastic modulus of permeable concrete when the porosity is 0 (N/m^2^); *m*_0_ is the coefficient to be determined; and *P_T_* is the total porosity of the permeable concrete (%).

The surface fracture energy of a material refers to the energy required to fracture the material. For permeable concrete, the internal part of the fracture that requires energy at the point of breakage only refers to the solid–solid joint. Therefore, the surface fracture energy of permeable concrete is also related to the total porosity, with the expression:

(6) $$\gamma =\gamma_{0}e^{-n_{0}P_{T}}$$

where $\gamma_{0}$ is the fracture energy of permeable concrete when the porosity is 0 (J), and *n*_0_ is the coefficient to be determined.

In Griffith fracture theory, α is half of the initial crack length. In permeable concrete, this value is related to the diameter of the pores. The equivalent diameter *d_p_* is used to measure the distribution of pore diameters within the permeable concrete [27], and the expression of the function of equivalent diameter is:

(7) $$\alpha =k_{0}d_{p}$$

Substituting these three expressions into the theoretical model and normalizing the equivalent diameter to a constant term, the expression for the ultimate stress at damage of the modified permeable concrete was obtained as follows:

(8) $$\delta_{max}=\sqrt{\frac{2E_{0}e^{-m_{0}P_{T}}\cdot \gamma_{0}e^{-n_{0}P_{T}}}{\pi \cdot k_{0}d_{p}}}=\sqrt{\frac{2E_{0}\gamma_{0}}{\pi k_{0}}}\cdot \frac{e^{-(m_{0}+n_{0})P_{T}}}{\sqrt{d_{p}}}=M_{0}\cdot \frac{e^{-OP_{T}}}{\sqrt{d_{p}}}=M\cdot e^{-OP_{T}}$$

**Figure 6:**
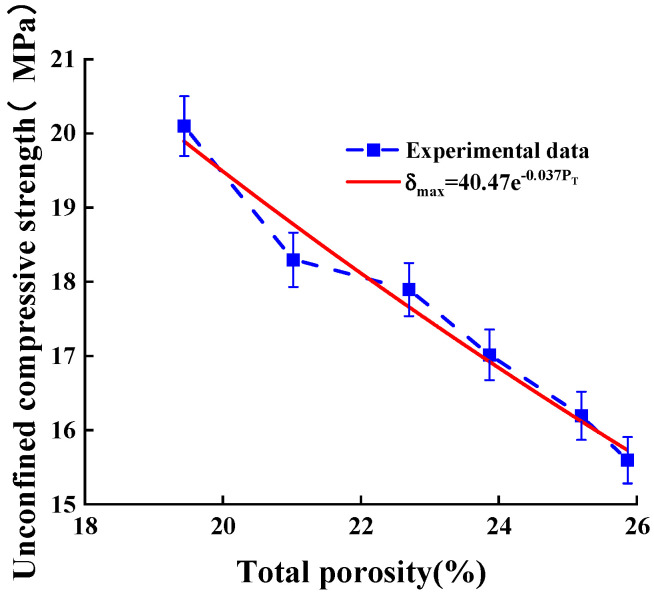
Compressive strength prediction model.

Based on this expression, the test data were fitted as shown in Figure 6.

This model was based on Griffith fracture theory, with the total porosity of permeable geopolymer concrete as the independent variable, and its prediction of compressive strength was established; its expression is shown in Figure 6. The *R*^2^ value is 0.972, and the corrected *R*^2^ value is 0.967 and the fitting is successful.

(9) $$\delta_{max}=40.47\cdot e^{-0.037P_{T}}$$

The total porosity and unconfined compressive strength are positively correlated in the range of the design porosity of the test block, and the relationship between the two is more clearly expressed on the basis of fracture theory. The *R*^2^ is more than 0.9, which indicates a high degree of fitting, and could provide a basis for predicting unconfined compressive strength through total porosity.

### 3.4. Effect of Fiber on Water Permeability and Porosity 

**Figure 7:**
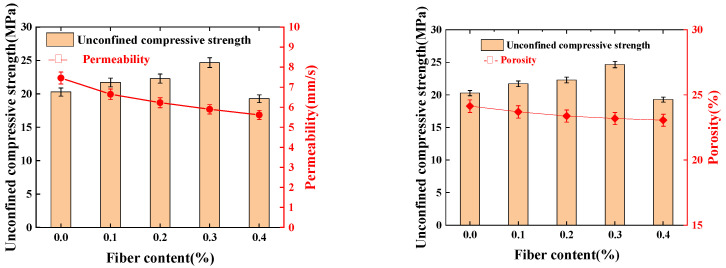
Plots of fibers’ influence on water permeability coefficient and porosity.

Figure 7 shows the relationship of fiber with the water permeability coefficient and porosity. From Figure 7, it can be seen that the water permeability coefficient and porosity of permeable concrete decrease with an increase in the content of fiber. On the one hand, this is because the mixing of fibers will occupy a part of the volume of the connected pores in the permeable concrete, resulting in an increase in the number of closed pores and effective drainage pore reduction; on the other hand, in a certain range of volume, fiber mixing will cause an increase in the number of “agglomerate” phenomena, which will block the pores. The blockage of pore space will lead to a decrease in porosity and the permeability coefficient. The change curves of the relationship of porosity and the permeability coefficient with fiber content are consistent; this is mainly because in the permeable concrete porosity test including connected pores and semi-connected pores, semi-connected pores for drainage cannot serve as direct channels for water circulation and can only temporarily store water.

As can be seen from Figure 7, the unconfined compressive strength of the specimen shows a trend of increasing first, and then, decreasing with an increase in fiber dosage, and the unconfined compressive strength is the largest when the dosage is 0.3%. The specific surface area of steel fiber is larger when the fiber content is larger, and the cement paste wrapped around the aggregate is reduced, so the amount of cementitious material is approximately the same; this results in thinning of the bonding layer between the aggregates, and the doping of excessive steel fiber weakens the bonding strength of the cementitious layer, which leads to a reduction in its strength.

### 3.5. Stress–Strain Relationship for Fiber-Reinforced Permeable Geopolymer Concrete

**Figure 8:**
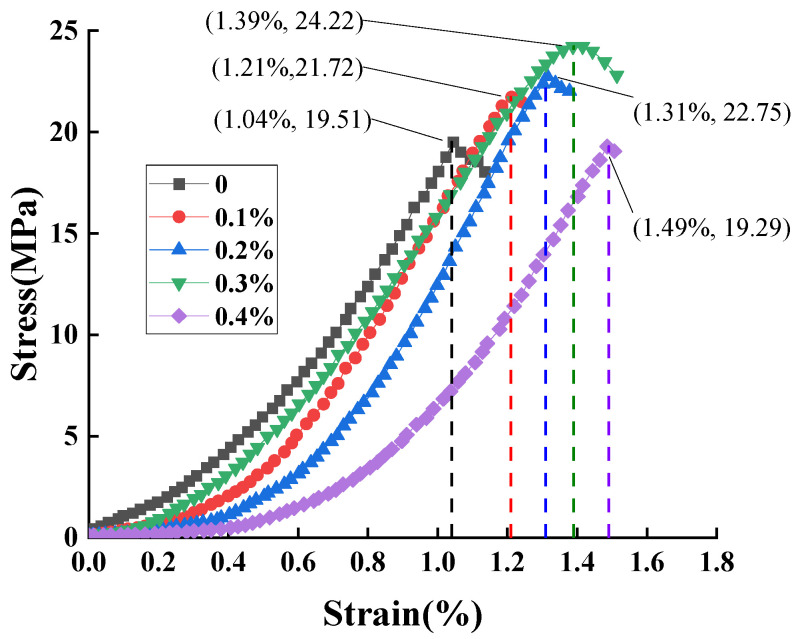
Stress–strain relationship.

Figure 8 shows stress–strain curves of the specimens with different fiber content, from which it can be seen that the unconfined compressive strength reaches a maximum of 24.22 MPa at a fiber content of 0.3%, which is 24.14% higher than that of the specimens without fiber. Compared with the low-polymer permeable concrete without fiber, the strains of the specimens after adding fiber are improved, and the strain reaches the maximum when the fiber content is 0.4%, and the maximum strain of the specimens after adding steel fiber is 43.27% higher than that of the specimens without steel fiber; this shows that the steel fiber improves the toughness of the permeable geopolymer concrete.

### 3.6. Strain Nephogram Analysis Based on Vic-3D Technology

**Figure 9:**
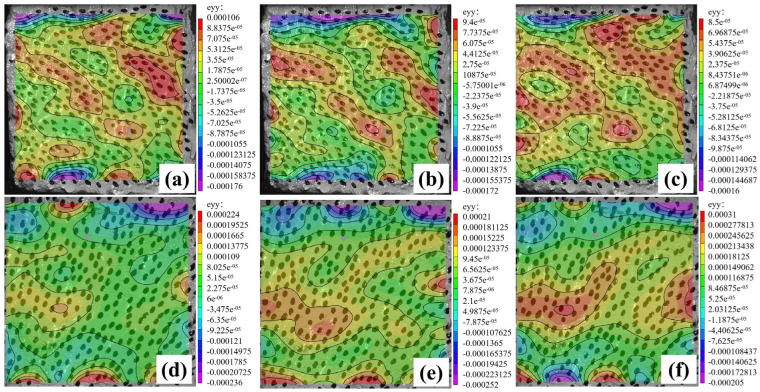
Vertical strain cloud diagram of permeable geopolymer concrete: (**a**) pre-loading of fiberless permeable geopolymer concrete; (**b**) mid-loading of fiberless permeable geopolymer concrete; (**c**) late loading of fiberless permeable geopolymer concrete; (**d**) pre-loading of fiber-reinforced permeable geopolymer concrete; (**e**) mid-loading of fiber-reinforced permeable geopolymer concrete; (**f**) late-loading of fiber-reinforced permeable geopolymer concrete.

Figure 9 shows a vertical strain nephogram of permeable geopolymer concrete during the loading process. From Figure 9a–c, it can be seen that with an increase in the load on the surface of the specimen, there is a diagonal strain concentration area, and the range also expands until it occupies the entire section. There is a clear destructive trend, and at this point, the specimen has been completely destroyed. From Figure 9d,e, it can be seen that with an increase in load, the surface of the specimen only produces an obvious strain concentration area in the middle. Fiberless permeable geopolymer concrete damage shows a greater stress concentration area. After adding steel fiber, the fiber permeable geopolymer concrete stress concentration area is less than that of the fiberless permeable geopolymer concrete, indicating that the addition of steel fiber can improve the toughness of permeable geopolymer concrete. Regarding strain, for the non-fiber-reinforced permeable geopolymer concrete in the early stage of loading on the strain concentration region, the test block damage from the strain concentration region gradually expands. For the fiber-reinforced permeable geopolymer concrete, with an increase in load, the strain region spreads to the non-strain concentration region, but the strain change is small, indicating that the fiber in the test block as a whole has the role of a tensile knot, which improves the mechanical properties of the test block.

## 4. Micro-Morphological Analysis of Fiber-Reinforced Permeable Geopolymer Concrete

### 4.1. Microanalysis of Slurry and Aggregate

**Figure 10:**
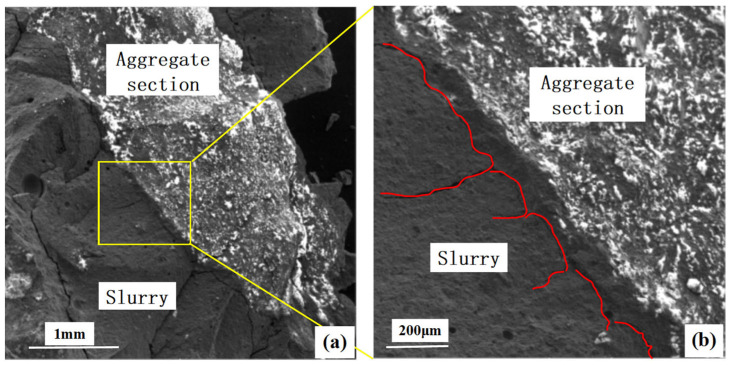
Scanning electron microscope images of the slurry–aggregate interface. (**a**) 71× Magnification (**b**) 245× Magnification.

Figure 10 shows scanning electron microscope images of the permeable geopolymer concrete after 28 days of maintenance and destruction, in the geopolymer colloid and aggregate and only in the geopolymer colloid, which have different densities, to produce micro-cracks. It can be seen that the filler effect of the metakaolin colloid is effectively exerted, and there is sufficient activity of Al_2_O_3_ and SiO_2_, hydration products of the Ca (OH)_2_ secondary hydration reaction, which effectively reduces pores and cracks in the structure.

### 4.2. Microanalysis of Fiber Permeable Geopolymer Concrete

**Figure 11:**
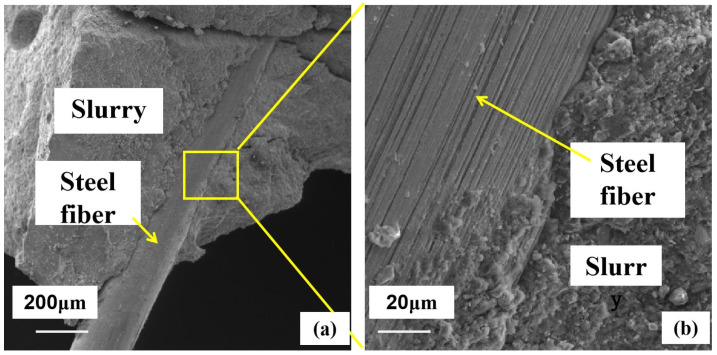
Scanning electron microscopy of fiber–plasma interface. (**a**) 200× Magnification (**b**) 2000× Magnification.

Figure 11 shows a scanning electron microscope image of the permeable geopolymer concrete mixed with steel fibers after damage. From Figure 11a, it can be seen that, overall, the fiber structure in the slurry is good, and when the specimen is damaged, some of the fibers are anchored in the slurry, indicating that the fibers and the slurry form a tight grip. With the fibers and slurry joints magnified 10 times, as shown in Figure 11b, it can be seen that the fibers and the slurry between the cracks are very small, and small amounts of metakaolin activation products are attached to the surface of the steel fibers, indicating that the bonding of the steel fibers and slurry is good. For the steel fibers with an increased specimen load on their surface, there are many parallel scratches, indicating that the test block in the destruction of the fibers here provides tensile force. After mixing the steel fiber, the fiber and the slurry, we can see the obvious role of fiber in the destruction of the tensile knot and an improvement in the test block’s bearing capacity. The appropriate amount of fiber can effectively improve the mechanical properties of permeable geopolymer concrete.

### 4.3. Energy-Dispersive Spectroscopy and X-ray Diffraction Analysis

**Figure 12:**
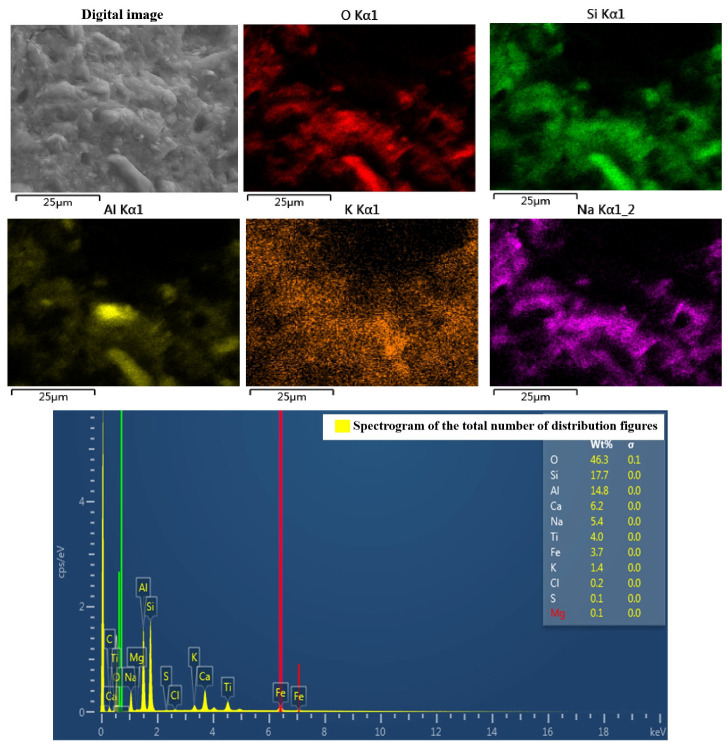
Plasma energy spectrum analysis.

**Figure 13:**
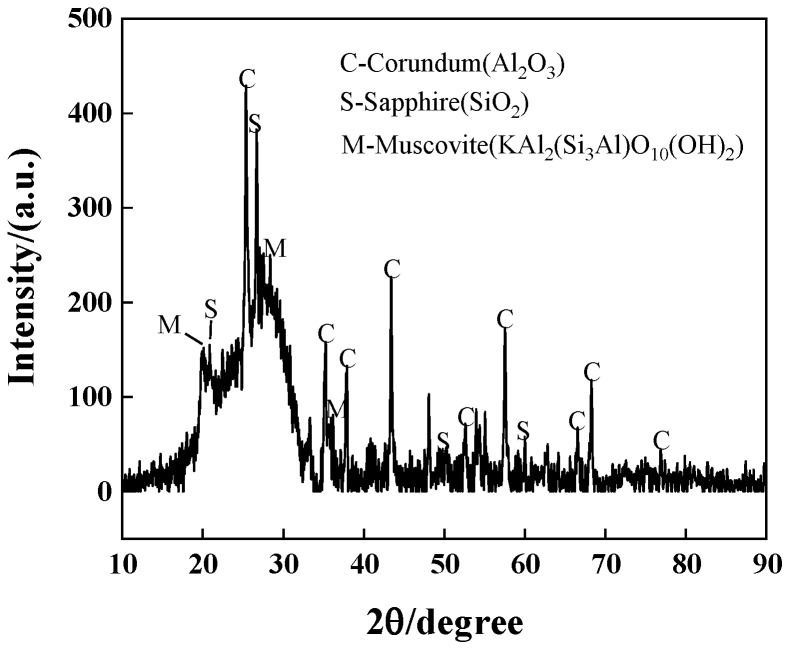
Slurry X-ray diffractogram.

Figure 12 shows the energy spectrum of the colloidal cross-section of the geopolymer, which mainly contains the elements Si, O and Al, and except for a small amount of Cl, O is the main anion. Thus, it can be seen that the metal elements and silicon should be present in the form of oxides, and correspond to the main components of kaolinite, Al_2_O_3_ and SiO_2_, with few other impurities. This represents the main area of geopolymer production. Combined with the X-ray diffractogram in Figure 13, it can be seen that there are also a large number of silica–oxygen components in the slurry, and the three-dimensional mesh structure of the geopolymer occurs because the aluminum–oxygen tetrahedra cannot complete the sharing of a bridging oxygen, which can only be shared by silica–oxygen tetrahedra or separately exist. So, the unsaturated oxygen in the structural unit constantly combines with other tetrahedra to enable three-dimensional expansion. As the reaction proceeds, the number of bridging oxygens becomes larger and the chain of silica–oxygen tetrahedra becomes relatively longer and denser in structure.

## 5. Conclusions

This study of steel fiber-reinforced permeable geopolymer concrete shows that at an alkali activator solution modulus of 1.4, the activation of metakaolin is the best; when the activation-to-solid ratio is 0.9, the metakaolin can be fully activated; the permeability coefficient of the prepared permeable geopolymer concrete is 7.46 mm/s, with a strength of 20.29 MPa, which is in line with the design specification of permeable concrete. At the same time, the chemical properties of the alkali-activated metakaolin products obtained through chemical analysis are relatively stable. Therefore, this study confirms that geopolymer cementitious materials are high-quality materials that can be used instead of cement for making permeable concrete, and provides new ideas for other subsequent applications of geopolymer materials.

In order to improve the brittleness of geopolymer cementitious materials, in this study, steel fibers are selected to improve the ductility of permeable geopolymer concrete, and signs of steel fibers providing load-bearing capacity at the point of specimen damage are observed from a microscopic point of view. By adding different quantities of steel fibers, the strength of the specimen is shown to increase and then decrease, and the specimen reaches optimum performance at an admixture volume of 0.3% of steel fibers. For the selection of fibers, subsequent studies could choose fibers from other materials to enhance the performance of permeable geopolymer concrete while better reducing the economic cost of production.

## Figures and Tables

**Table 1:** Components of metakaolin (wt.%).

SiO_2_	Al_2_O_3_	Fe_2_O_3_	TiO_2_	CaO	MgO	K_2_O	Na_2_O
55.06	43.02	0.76	0.24	0.17	0.68	0.55	0.06

**Table 2:** Basic properties of soluble glass.

SiO_2_ (wt.%)	Na_2_O (wt.%)	Density Be/20 °C	Modulus (M)
37.3	8.54	38.5 Be	3.3

**Table 3:** Performance indexes of naphthalene water-reducing agents.

Solid	pH	Water Reduction Rate	Na_2_SO_4_
≥92	7~9	12~20 wt.%	16~19 wt.%

## Data Availability

The data used to support the findings of this study are available from the corresponding author upon request.
